# Improving Electroluminescence Efficiency by Linear Polar Host Capable of Promoting Horizontal Dipole Orientation for Dopant

**DOI:** 10.1002/advs.202206420

**Published:** 2022-12-25

**Authors:** Maoxing Yu, Xing Wu, Hao Liu, Zuguo Yang, Nuoling Qiu, Dezhi Yang, Dongge Ma, Ben Zhong Tang, Zujin Zhao

**Affiliations:** ^1^ State Key Laboratory of Luminescent Materials and Devices Guangdong Provincial Key Laboratory of Luminescence from Molecular Aggregates South China University of Technology Guangzhou 510640 China; ^2^ School of Science and Engineering Shenzhen Institute of Aggregate Science and Technology The Chinese University of Hong Kong Shenzhen Guangdong 518172 China

**Keywords:** dipole‐dipole interaction, horizontal dipole orientation, host materials, light out‐coupling, organic light‐emitting diodes

## Abstract

In doped organic light‐emitting diodes (OLEDs), the host materials play an important role in emitting layers. Most studies about host materials mainly focus on their energy levels and carrier transport behaviors, while less attention is paid to their influence on the dipole orientation of dopants, which closely associate with the light out‐coupling efficiency (*η*_out_) of the device. Herein, a linear polar host material (*l*‐CzTRZ) consisting of carbazole donor, triazine acceptor, and the conjugated *para*‐terphenyl skeleton is developed and its crystal and electronic structures, thermal and electrochemical stabilities, optical property, and carrier transport ability are investigated. *l*‐CzTRZ prefers ordered horizontal orientation and favors electron transport in neat film. More importantly, it can promote horizontal dipole orientation for the dopants via dipole‐dipole interaction, furnishing an excellent horizontal dipole ratio of 91.5% and thus a high *η*_out_ of 43% for the phosphorescent dopant (PO‐01‐TB). Consequently, the OLED with *l*‐CzTRZ host and PO‐01‐TB dopant attains state‐of‐the‐art electroluminescence efficiencies of 135.5 cd A^−1^, 135.7 lm W^−1^ and 41.3%, with a small roll‐off of 9.7% at 5000 cd m^−2^ luminance. The presented significant impact of the host on the dipole orientation of the dopant shall enlighten the design of host materials to improve OLED performance.

## Graphical abstract

A linear polar host material with high thermal and electrochemical stabilities and electron‐dominated carrier transport ability can promote horizontal dipole orientation for phosphorescent dopants via dipole‐dipole interaction, furnishing ultrahigh electroluminescence efficiencies of up to 41.3% with small roll‐offs.

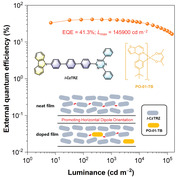

## 1 Introduction

Organic light‐emitting diodes (OLEDs) with competitive advantages of flexibility, low power consumption, high color gamut, wide viewing angle, etc., have developed rapidly and occupied a considerable market of display panels.^[^ ^1^ ^]^ Luminescent materials are the essential components responsible for converting electric energy to photons in OLEDs. So far, many kinds of luminescent materials including fluorescent materials, phosphorescent materials as well as those with thermally activated delayed fluorescence have been explored and extensively studied.^[^ ^2^ ^]^ The most popular and successfully commercialized luminescent materials for OLEDs are phosphorescent materials, owing to their theoretical 100% utilization of electro‐generated excitons via intersystem crossing facilitated by heavy atom effect of the noble metals.^[^ ^3^ ^]^ However, in most cases, phosphorescent materials encounter thorny problems of emission quenching and exciton annihilation at high concentrations or in an aggregate state. So, host materials are needed to disperse phosphorescent molecules to alleviate this situation.^[^ ^4^ ^]^ In addition to suppress the accumulation of luminescent molecules, certain functional host materials, such as bipolar hosts and exciplex hosts, are able to balance carrier transport and increase exciton harvesting to optimize device performance.^[^ ^5^ ^]^ Therefore, the creation of robust and efficient host materials also plays a critical role in the advancement of OLEDs.

**Figure 1:**
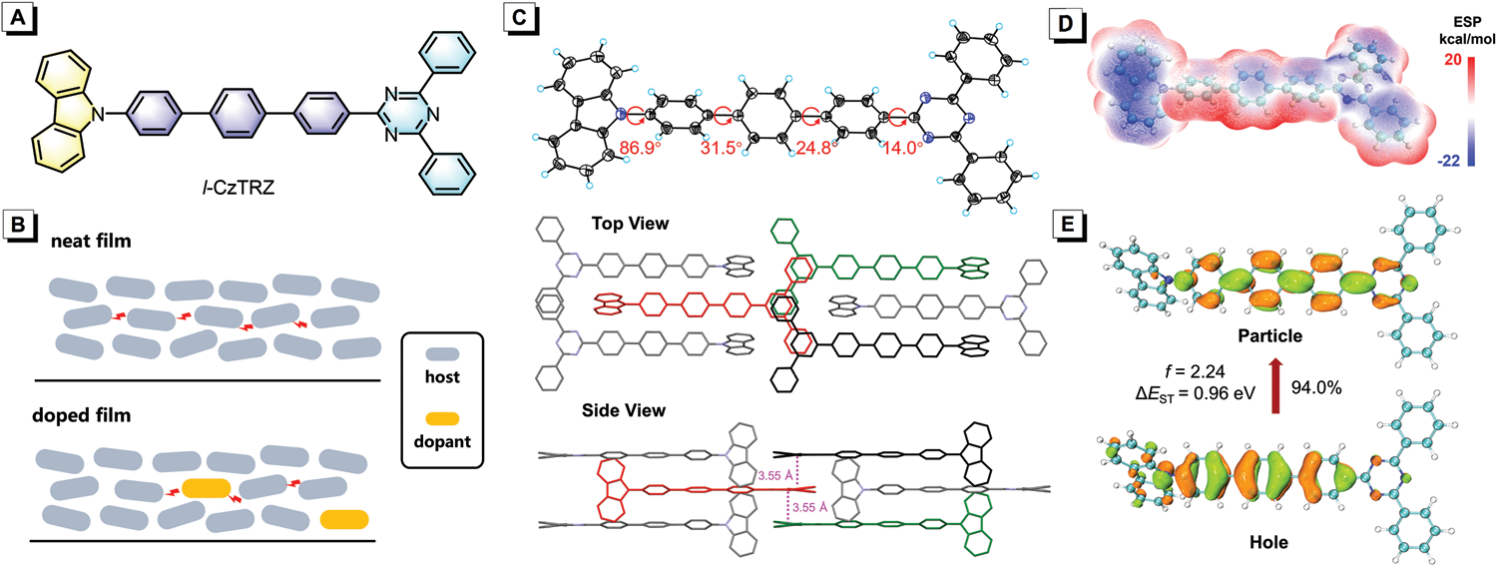
A) Molecular structure of *l*‐CzTRZ. B) The interactions of host and dopant in neat and doped films. C) Single‐crystal structure and packing pattern of *l*‐CzTRZ (CCDC 21 31 603). D) Electrostatic potential surface distribution of *l*‐CzTRZ calculated based on the crystal structure. E) The natural transition orbitals of the S_1_ state of *l*‐CzTRZ.

In the design of host materials, high thermal stability, balanced bipolar carrier transport, and high triplet energy level (*E*_T_) are commonly regarded as important factors to improve exciton recombination, encourage energy transfer to luminescent dopants, and finally achieve high electroluminescence (EL) efficiencies.^[^ ^6^ ^]^ Actually, as another critical factor that positively associates with EL efficiencies, light out‐coupling efficiency (*η*_out_), should also be taken into consideration for host materials for maximizing device performance. However, the studies about *η*_out_ generally focus on luminescent materials by horizontal dipole orientation engineering on their molecular structures.^[^ ^7^ ^]^ Less attention is paid to the host materials that can induce horizontal dipole orientation for ordinary luminescent dopants,^[^ ^8^ ^]^ although it could be a more facile and versatile strategy to enhance *η*_out_s of the devices. Recent research shows that the dipole–dipole interactions between the polar dopant and polar host contribute to molecular ordering during the vacuum‐deposition of thin films.^[^ ^8c^ ^]^ Certain exciplex host systems with high polarity were reported to be beneficial for improving horizontal dipole ratios (*Θ*_//_s) and thus *η*_out_s of the dopants, but the combination is complicated and the device stability is not satisfactory in many cases. Therefore, in this contribution, a new linear polar host (*l*‐CzTRZ) consisting of a carbazole donor, triazine acceptor, and the strongly conjugated *para*‐terphenyl skeleton is designed and prepared (**Figure** **1**A). *l*‐CzTRZ is highly thermal stability and shows excellent electron‐transporting capacity. More importantly, it can promote horizontal dipole orientation for phosphorescent dopants via dipole–dipole interaction (Figure 1B), providing an ultrahigh external quantum efficiency (*η*_ext_) of 41.3%.

## 2 Results and Discussion

*l*‐CzTRZ was facilely synthesized through a one‐step Suzuki reaction as shown in Scheme S1 (Supporting Information), and purified via column chromatography, recrystallization, and subsequent temperature‐gradient vacuum sublimation. The structure was characterized by NMR, high‐resolution mass spectrometry as well as crystallography analysis on single crystals obtained by sublimation. The crystal structure shows that *l*‐CzTRZ holds a linear structure, in which carbazole is attached to the *para*‐terphenyl in a highly twisted manner with a large torsional angle of 86.9°, while triazine is connected to *para*‐terphenyl in a quiet planar manner with a small torsional angle of 14.0°. *l*‐CzTRZ adopts regular and parallel packing in crystals, and close *π*–*π* stacking is observed among triazine groups with inter‐plane distances of ≈3.55 Å (Figure 1C). Such kind of tight *π*–*π* stacking with a large overlap and strong interactions among triazine moieties that govern electron transport is conducive to improving the electron‐transporting ability of *l*‐CzTRZ.^[^ ^6e^ ^]^

**Figure 2:**
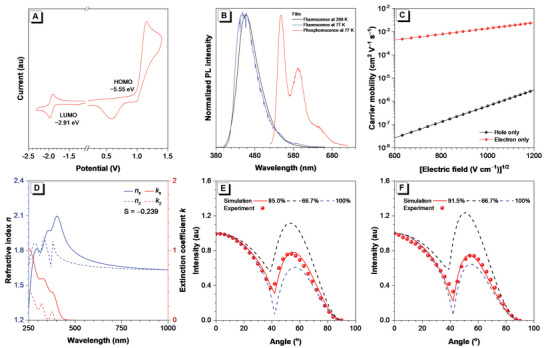
A) Cyclic voltammogram of *l*‐CzTRZ. B) Normalized fluorescence and phosphorescence spectra of *l*‐CzTRZ neat film. C) Electric field–dependent carrier mobility of *l*‐CzTRZ neat film. D) Anisotropies in the refractive indices (blue) and the extinction coefficients (red) of *l*‐CzTRZ neat film. The solid and dash lines indicate horizontal and vertical components of optical constants, respectively. The *p*‐polarized PL intensity of E) *l*‐CzTRZ neat film and F) the doped film of 3 wt.% PO‐01‐TB: *l*‐CzTRZ as a function of the emission angle.

*l*‐CzTRZ has a high decomposition temperature of 464 °C, while no glass‐transition temperature is detected (Figure S1, Supporting Information), revealing *l*‐CzTRZ possesses excellent thermal stability, which is desired for the application as functional material in OLEDs. *l*‐CzTRZ also enjoys good electrochemical stability as evidenced by the reversible oxidation and reduction waves in cyclic voltammetry measurement (**Figure** **2**A). Its energy levels of the highest occupied molecular orbital and the lowest unoccupied molecular orbital are calculated as −5.55, and −2.91 eV, respectively.

To study the electronic structure, theoretical calculations based on time‐dependent density functional theory simulations with M06‐2X/6‐31G** are performed.^[^ ^9^ ^]^ The natural transition orbitals of the ground state to the lowest singlet excited state (S_0_→S_1_) transition show that most of the hole and particle distributions are concentrated on the *para*‐terphenyl skeleton, presenting obvious local excitation (LE) characteristic (Figure 1E). The good conjugation of the linear skeleton endows *l*‐CzTRZ with a large S_0_→S_1_ oscillator strength (2.24). The calculated energy levels of S_1_ state and the lowest triplet excited (T_1_) state is 3.23 and 2.27 eV, respectively, corresponding to an adiabatic energy splitting between S_1_ and T_1_ states (Δ*E*_ST_) of 0.96 eV. The electrostatic potential (ESP) distribution^[9a^ ^]^ calculated based on a single crystal structure (Figure 1D) shows that the electronegative and electropositive regions are basically distributed on the two orthogonal directions, tending to interact with neighboring molecules as much as possible by large overlap. When doped with dopants, the hosts can interact with the dopants and influence their alignment through electrostatic interaction. The static dipole moment is calculated to be 2.4 Debye for *l*‐CzTRZ, indicative of relatively high molecular polarity, which also plays an important role in intermolecular dipole‐dipole interaction. Moreover, the molecular polarity can also be evaluated by using the molecular polarity index (MPI) based on ESP.^[^ ^9b^ ^]^ It is found that *l*‐CzTRZ has a large MPI value of 8.3 kcal mol^−1^, further confirming the relatively high polarity, which can contribute to the molecular ordering during deposition and influence the alignment of the dopants.^[^ ^8c^ ^]^

*l*‐CzTRZ exhibits an absorption maximum at 342 nm in toluene, assigned to the absorption of the *π*–π* transition of the conjugated molecular backbone. Owing to the obvious LE characteristic, *l*‐CzTRZ has a short‐wavelength photoluminescence (PL) peak at 416 nm in toluene (Figure S2, Supporting Information), with a high PL quantum yield (*Φ*_PL_) of 90.4%. In the neat film, the PL peak of *l*‐CzTRZ is red‐shifted to 455 nm with a decreased *Φ*_PL_ of 62.1%, due to strong *π*–*π* interaction. *l*‐CzTRZ shows short PL lifetimes of 1.3 and 18.1 ns in toluene and neat film, respectively, and no obvious delayed components are observed in transient PL decay curves (Figure S3, Supporting Information). The Δ*E*_ST_ of *l*‐CzTRZ in the neat film is calculated to be as large as 0.63 eV from the fluorescence and phosphorescence spectra measured at 77 K (Figure 2B), which is unfavored for reverse intersystem crossing. Therefore, *l*‐CzTRZ has no delayed fluorescence feature and exhibits normal fluorescence property. The *E*_T_ of *l*‐CzTRZ in the neat film is calculated as 2.34 eV from the onset of the phosphorescence spectrum.

To investigate the potential of *l*‐CzTRZ as a host material for OLEDs, its carrier transport behavior is studied by the space charge limited current method.^[^ ^10^ ^]^ Hole‐ and electron‐only devices with configurations of ITO/TAPC (10 nm)/*l*‐CzTRZ (80 nm)/TAPC (10 nm)/Al and ITO/TmPyPB (10 nm)/*l*‐CzTRZ (80 nm)/TmPyPB (10 nm)/LiF (1 nm)/Al are fabricated, respectively, in which 1,1‐bis[(di‐4‐tolylamino)phenyl]cyclohexane (TAPC) and 1,3,5‐tri(m‐pyrid‐3‐yl‐phenyl)benzene (TmPyPB) are used as buffer layers. Thanks to the tight *π*–*π* stacking of triazine moieties, *l*‐CzTRZ displays an electron‐dominated carrier transport feature. For example, at an electric field of 3.6 × 10^5^ V cm^−1^, the electron mobility of *l*‐CzTRZ is 4.38 × 10^−4^ cm^2^ V^−1^ s^−1^, being more than four orders of magnitude larger than hole mobility (2.69 × 10^−8^ cm^2^ V^−1^ s^−1^).

**Figure 3:**
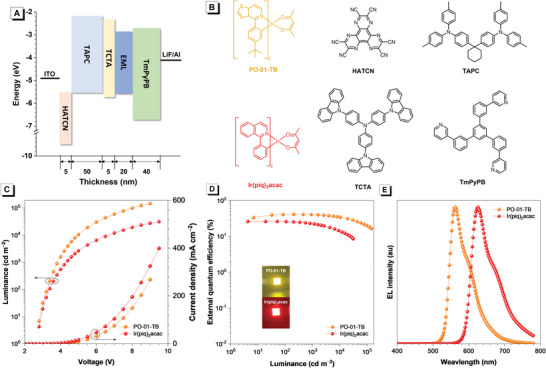
A) Device architecture and energy diagram. B) Structures of the phosphorescent emitters and other functional materials for the vacuum‐deposited OLEDs. Plots of C) luminance–voltage–current density, D) external quantum efficiency−luminance and E) EL spectra at 1000 cd m^−2^ of PhOLEDs based on the *l*‐CzTRZ host.

Then, the performance of *l*‐CzTRZ as a host material for phosphorescent OLEDs (PhOLEDs) is investigated, and the orange PO‐01‐TB and red Ir(piq)_2_acac are selected as phosphorescent dopants in view of the relatively low *E*_T_ of *l*‐CzTRZ. The PhOLEDs with a configuration of ITO/HATCN (5 nm)/TAPC (50 nm)/TCTA (5 nm)/EML (20 nm)/TmPyPB (40 nm)/LiF (1 nm)/Al are fabricated (**Figure** **3**A), in which EML is composed of a doped film of 3 wt.% PO‐01‐TB or 3 wt.% Ir(piq)_2_acac in the *l*‐CzTRZ host, hexaazatriphenylenehexacabonitrile (HATCN) serves as hole injection layer, TAPC functions as a hole‐ transporting layer, tris(4‐(carbazol‐9‐yl)phenyl)amine (TCTA) works as an electron‐blocking layer, and TmPyPB behaviors as an electron‐transporting layer (Figure 3B). The obtained PhOLEDs have low turn‐on voltages of 2.7 V, indicative of efficient injection and transport of the carriers. The devices of PO‐01‐TB and Ir(piq)_2_acac show EL peaks at 562 and 626 nm, respectively, which are consistent with the PL peaks of these phosphorescent dopants, without the appearance of the emission from *l*‐CzTRZ. Both PhOLEDs can provide impressive EL performances, with large luminance and high efficiencies (**Table** **1**). Delightfully, the orange device of PO‐01‐TB exhibits remarkable EL performance, and the maximum luminance (*L*), current efficiency (*η*_C_), power efficiency (*η*_P_), and external quantum efficiency (*η*_ext_) are as high as 145 900 cd m^−2^, 135.5 cd A^−1^ and 135.7 lm W^−1^ and 41.3%, respectively. The EL efficiencies are maintained at 122.7 cd A^−1^ and 85.4 lm W^−1^ and 37.3%, respectively, even at high luminance of 5000 cd m^−2^. In addition, the red device of Ir(piq)_2_acac also exhibits excellent luminance and EL efficiencies of 31 200 cd m^−2^, 21.3 cd A^−1^, 23.7 lm W^−1,^ and 26.1%, with a small roll‐off of 13.2% at 1000 cd m^−2^ (Figure 3C,D).

**Table 1:** EL performances of PhOLEDs using *l*‐CzTRZ as host.^a)^

EML	*V*_on_ [V]	*η*_C_ [cd A^−1^]	*η*_P_ [lm W^−1^]	*η*_ext_ [%]	*L*_max_ [cd m^−2^]	CIE (x,y)	*λ*_EL_ [nm]	RO [%]
3 wt.% PO‐01‐TB: *l*‐CzTRZ	2.7	135.5/133.3/122.7	135.7/109.3/85.4	41.3/40.6/37.3	145 900	(0.507, 0.241)	562	1.8/9.7
3 wt.% Ir(piq)_2_acac: *l*‐CzTRZ	2.7	21.3/18.6/14.5	23.7/13.6/8.1	26.1/22.6/17.2	31 200	(0.676, 0.321)	626	13.2/33.9

^a)^ Abbreviations: *V*_on_ = turn‐on voltage at 1 cd m^−2^; *η*_C_ = current efficiency of maximum value/at 1000 cd m^−2^/ at 5000 cd m^−2^; *λ*_EL_ = EL peak; *η*_P_ = power efficiency of maximum value/at 1000 cd m^−2^/ at 5000 cd m^−2^; *λ*_EL_ = EL peak; *η*_ext_ = external quantum efficiency of maximum value/at 1000 cd m^−2^/ at 5000 cd m^−2^; *L*_max_ = maximum luminance; CIE = Commission Internationale de I'Eclairage coordinates; *λ*_EL_ = EL peak; RO: *η*_ext_ roll‐off at 1000 cd m^−2^ and 5000 cd m^−2^. EML = emitting layer.

To understand the outstanding *η*_ext_ exceeding 30% of PO‐01‐TB, the *Θ*_//_ of the doped film of 3 wt.% PO‐01‐TB: *l*‐CzTRZ is measured by using the angle‐dependent *p*‐polarized PL spectra. The result shows that the *Θ*_//_ of the doped film is as large as 91.5%. Consequently, an excellent *η*_out_ of 43% is calculated based on *Θ*_//_, and the thickness and reflex index of active layers of the device, accounting for the remarkably high *η*_ext_. Besides, the doped film of 3 wt.% Ir(piq)_2_acac: *l*‐CzTRZ also shows a high *Θ*_//_ of 83.0% (Figure S5A, Supporting Information). However, according to the references, similar iridium complexes doped in common hosts only show low or moderate *Θ*_//_ values, normally less than 75%.^[^ ^8a^ ^]^ Further, the widely used low‐polar linear host material of 4,4′‐bis(*N*‐carbazolyl)‐1,1′‐biphenyl (CBP) is used as a control host material for PO‐01‐TB, as its analogous linear configuration is like to align horizontally. By doping 3 wt.% PO‐01‐TB in the CBP host, the *Θ*_//_ is measured as 76% (Figure S5B, Supporting Information), close to the values of many iridium complexes in the CBP host, but much lower than that in the *l*‐CzTRZ host, indicating *l*‐CzTRZ can function much better than CBP to promote horizontal dipole orientation for the dopant. In addition, the dipole orientation of the dopants in a polar analog containing a biphenyl linker and the same functional units of *l*‐CzTRZ, 9‐(4’‐(4,6‐diphenyl‐1,3,5‐triazin‐2‐yl)biphenyl‐4‐yl)‐9H‐carbazole, is further studied. It is found that this polar analog can promote horizontal dipole orientation of the dopants as well, leading to high *Θ*_//_ values of 85% and 80% for PO‐01‐TB and Ir(piq)_2_acac, respectively (Figure S6, Supporting Information). Both *Θ*_//_ values are higher than those in a low‐polar CBP host, indicating the polar property of the host can provide a conducive environment for dipole‐dipole interaction to facilitate horizontal dipole orientation for the dopants.

Variable‐angle spectroscopic ellipsometry (VASE) measurement is capable of characterizing the average orientation of the absorbing transition dipole moment and the results are suitable to analyze molecular orientation in pure films.^[^ ^8b^ ^]^ To decipher the interesting phenomenon, VASE is performed to survey the molecular alignment of *l*‐CzTRZ in neat film (Figure 2D). As a result, *l*‐CzTRZ exhibits an orientation order parameter (*S*) of −0.239, estimated from the largest absorption peak, which is between the values of completely random orientation (*S* = 0) and completely horizontal orientation (*S* = −0.5).^[^ ^7a^ ^]^ Furthermore, angle‐dependent *p*‐polarized PL spectra indicate that the transition dipole moment of the *l*‐CzTRZ neat film also has a good *Θ*_//_ value of 85.0% (Figure 2E), higher than the value for random dipole orientation (66.7%). So, these findings disclose that *l*‐CzTRZ has an anisotropic characteristic of horizontal dipole oritentation in neat film, which can exert a significant impact over the orientation of lowly doped PO‐01‐TB by dipole‐dipole interaction,^[^ ^8b,c^ ^]^ This is also true for 9‐(4’‐(4,6‐diphenyl‐1,3,5‐triazin‐2‐yl)biphenyl‐4‐yl)‐9H‐carbazole, but such effect is inferior in low‐polar CBP host. In a word, the polar characteristic and ordered horizontal dipole orientation of *l*‐CzTRZ can effectively promote the horizontal dipole orientation for PO‐01‐TB dopant via dipole‐dipole interaction, which eventually greatly improves the *η*_out_ and thus *η*_ext_ of PO‐01‐TB.

## 3 Conclusion

In summary, a linear rigid host *l*‐CzTRZ comprised of carbazole donor, triazine acceptor, and conjugated *para*‐terphenyl backbone is developed. *l*‐CzTRZ has high thermal and electrochemical stabilities and possesses high polarity. It prefers ordered horizontal orientation and favors electron transport, as evidenced by its fast electron mobility, being more than four orders of magnitude larger than its hole mobility in neat film, owing to the close *π*–*π* stacking interaction among triazine moieties. *l*‐CzTRZ can serve as a promising host material for orange and red phosphorescent emitters, providing high EL efficiencies and very small roll‐offs. Impressively, the PhOLED with PO‐01‐TB dopant and *l*‐CzTRZ host can radiate strong orange light with a high maximum luminance of 145 900 cd m^−2^, and outstanding *η*_C_, *η*_P_ and *η*_ext_ of 135.5 cd A^−1^, 135.7 lm W^−1^ and 41.3%, respectively, with a small efficiency roll‐off of 9.7% even at high luminance of 5000 cd m^−2^, which are much superior to the reported data for PO‐01‐TB. The main cause for this greatly improved EL performance of PO‐01‐TB is that *l*‐CzTRZ can promote horizontal dipole orientation for PO‐01‐TB via dipole‐dipole interaction, leading to a high *Θ*_//_ of 91.5% and thus an excellent *η*_out_ of 43%. These findings demonstrate that exploring robust polar hosts that can induce horizontal dipole orientation for dopants should be a feasible and versatile approach for realizing high‐performance OLEDs.

## Conflict of Interest

The authors declare no conflict of interest.

## Supporting information

Supporting Information

## Data Availability

The data that support the findings of this study are available from the corresponding author upon reasonable request.
