# Supplementary material for: Improving Electroluminescence Efficiency by Linear Polar Host Capable of Promoting Horizontal Dipole Orientation for Dopant
Source: Adv Sci (Weinh). 2022 Dec 25;10(6):2206420. doi: 10.1002/advs.202206420 (PMC9951345; doi:10.1002/advs.202206420)
Supplement: Supplementary file 1 — Supporting Information [file ADVS-10-2206420-s001.pdf]

Table of Contents

1. **General information** 2
2. **Synthesis and characterization** 2
   Scheme S1. Synthetic route of *l*-CzTRZ. 2
3. **X-ray crystallography** 3
4. **Thermal property** 3
   Figure S1. Thermogravimetric analysis and differential scanning calorimetry curves. 3
5. **Photophysical property** 3
   Figure S2. Absorption and nomalized PL spectra of *l*-CzTRZ in toluene (10^{-5} M). 3
   Figure S3. Transient PL decay spectra of *l*-CzTRZ in (A) toluene and (B) neat film. 4
   Figure S4. Fluorescence and phosphorescence spectra of *l*-CzTRZ in toluene, measured at 77 K. 4
5. **Horizontal dipole orientation** 4
   Figure S5. The *p*-polarized PL intensity of doped films 4
   Figure S6. The *p*-polarized PL intensity of the films associated with the linear polar analogue 5
6. **OLED fabrication and measurement** 5
7. **Reference** 5

1. General information

All the chemicals and reagents were purchased from commercial sources and used as received without further purification. ^{1}H NMR spectrum was measured on a Bruker AV 400 spectrometer in CD_2Cl_2 at room temperature. High resolution mass spectrum (HRMS) was recorded on a GCT premier CAB048 mass spectrometer operating in MALDI-TOF mode. Single crystal X-ray diffraction intensity data were collected on a Bruker–Nonices Smart Apex CCD diffractometer with MoKα radiation and a Rigaku XtaLAB P2000 FR-X with CuKα radiation. UV-vis absorption spectrum was measured on a Shimadzu UV-2600 spectrophotometer. PL spectra were recorded on a Horiba Fluoromax-4 spectrofluorometer. PL quantum yields were measured using a Hamamatsu absolute PL quantum yield spectrometer C11347 Quantaurus_QY. Transient PL decay spectra were measured using Quantaurus-Tau fluorescence lifetime measurement system (C11367-03, Hamamatsu Photonics Co., Japan). Thermogravimetric analyses (TGA) were performed on a TA Instruments TGA 5500. Differential scanning calorimetry (DSC) analysis was carried out on a TA Instruments DSC 2500. Cyclic voltammetry (CV) was performed on a CHI 610EA14297 in a solution of tetra-*n*-butylammonium hexafluorophosphate (Bu_4NPF_6) (0.1 M) in dichloromethane or dimethylformamide at a scan rate of 100 mV s^{-1}, using a platinum wire as the auxiliary electrode, a glass carbon disk as the working electrode and Ag/Ag$^+$ as the reference electrode. HOMO and LUMO energy levels are determined by the formula of HOMO = $-[E_{ox} - E_{1/2}(Fc/Fc^+) + 4.8]$ eV, and LUMO = $-[E_{re} - E_{1/2}(Fc/Fc^+) + 4.8]$ eV. E_{ox} and E_{re} represent the onset oxidation and reduction potentials relative to ferrocene, respectively. For the measurement of orientation of emitting dipoles in molecules, a setup RSQX-01 made by the Changchun Ruoshui Technology Development Co., Ltd. was used. The dipole orientation of the film was determined by angle-resolved and polarization resolved PL on a half quartz cylinder prism. A continuous-wave He:Cd laser (325 nm) with a fixed angle of 45° to the substrate was employed as excitation source. *p*-Polarized emitted light was detected at the respective peak wavelength of the PL spectrum of each film. The ground-state geometries were optimized by density functional theory (DFT) method and the photophysical properties of excited states were studied by time-dependent density functional theory (TDDFT). Geometry optimizations were performed employing M06-2X and the basis set 6-31G(d,p), treating crystal structure as the initial configuration. In TDDFT calculations, the optimized geometry structure was treated as initial configuration. Multiwfn was used to generate NTOs and the transition density matrix. DFT and TDDFT calculations were carried out in the Gaussian 16 package.

2. Synthesis and characterization

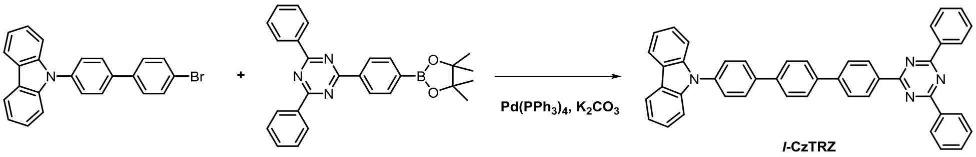

Scheme S1. Synthetic route of *l*-CzTRZ.

9-(4''-(4,6-Diphenyl-1,3,5-triazin-2-yl)-[1,1':4',1''-terphenyl]-4-yl)-9*H*-carbazole (*l*-CzTRZ): A mixture of 9-(4'-bromo-[1,1'-biphenyl]-4-yl)-9*H*-carbazole (6.0 g, 15.0 mmol), 2,4-diphenyl-6-(4-(4,4,5,5-tetramethyl-1,3,2-dioxaborolan-2-yl)phenyl)-1,3,5-triazine (5.9 g, 13.6 mmol), $Pd(PPh_3)_4$ (0.47 g, 0.41 mmol), K_2CO_3 (5.7 g, 40.9 mmol) was added in 250 mL two-neck bottle under nitrogen. Then, a mixed solvent system (80 mL) of THF and H_2O (v/v = 8:1) was injected into the bottle, and the reaction mixture was refluxed for 12 h. After cooling to room temperature, the mixture was poured into water and extracted twice with dichloromethane. The combined organic layers were washed with water and then dried over anhydrous magnesium sulfate. After filtration, the solvent was evaporated under reduced pressure and the residue was purified by silica gel column chromatography (petroleum ether/dichloromethane, 1:15). And then the obtained solid was recrystallization by dichloromethane. White solid of *l*-CzTRZ was obtained in 65% yield. ^{1}H NMR (400 MHz, CD_2Cl_2, δ) 8.92 (d, J = 8.2 Hz, 2H), 8.84–8.82 (m, 4H), 8.18 (d, J = 7.7 Hz, 2H), 7.96–7.87 (m, 8H), 7.72–7.61 (m, 8H), 7.53–7.44 (m, 4H), 7.32 (m, 2H). HRMS ($C_{45}H_{30}N_4$): m/z 627.2549 (M + H$^+$, calcd 627.2548). The ^{13}C NMR data of *l*-CzTRZ were not available due to its poor solubility.

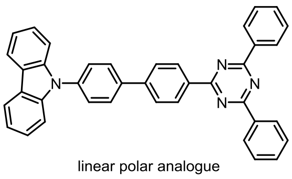

linear polar analogue

The linear polar analogue (9-(4'-(4,6-diphenyl-1,3,5-triazin-2-yl)biphenyl-4-yl)-9H-carbazole) was prepared according to the reference method.[1]

3. X-ray crystallography

Crystal data for *l*-CzTRZ (CCDC 2131603): $C_{45}H_{30}N_4$, M_W = 626.73, orthorhombic, P b c n, a = 12.6591(2) Å, b = 23.9957(4) Å, c = 10.6954(2) Å, α = 90°, β = 90°, γ = 90°, V = 3248.88(10) Å^3, Z = 4, D_c = 1.281 g cm^{-3}, μ = 0.586 mm^{-1} (CuKα, λ = 1.54184), F(000) = 1312, T = 150.00(10) K, 2θ_{max} = 67.051^o (99.9%), 12523 measured reflections, 2910 independent reflections (R_{int} = 0.0209), GOF on F^2 = 1.068, R_1 = 0.0376, wR_2 = 0.0963 (all data), Δe 0.159 and –0.273 eÅ^{-3}.

4. Thermal property

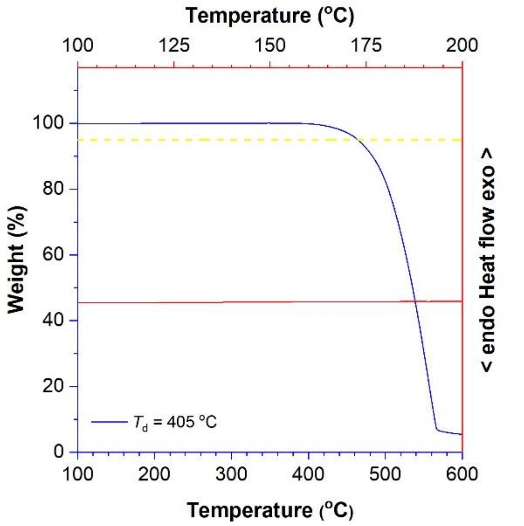

Figure S1. Thermogravimetric analysis and differential scanning calorimetry curves at a heating rate of 10 °C min^{-1} under nitrogen.

5. Photophysical property

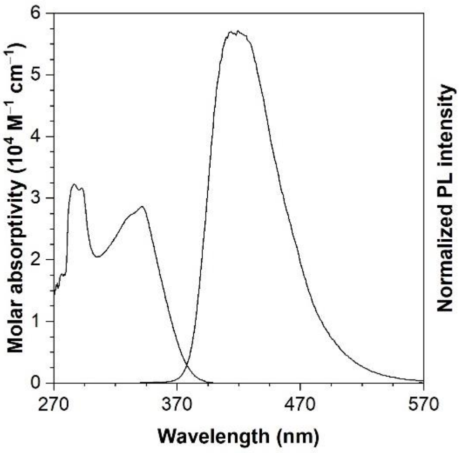

Figure S2. Absorption and nomalized PL spectra of *l*-CzTRZ in toluene (10^{-5} M).

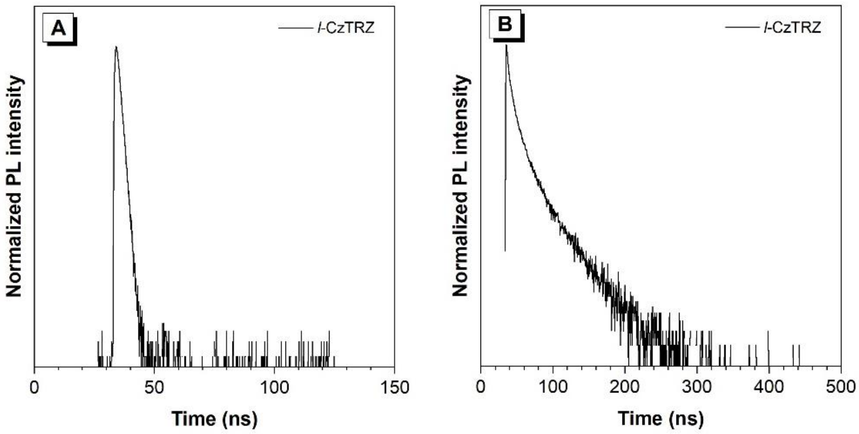

Figure S3. Transient PL decay spectra of *l*-CzTRZ in (A) toluene and (B) neat film.

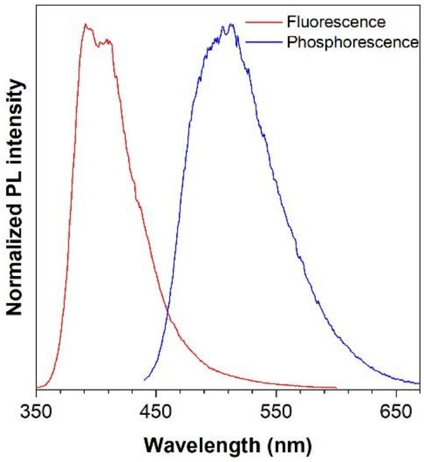

Figure S4. Fluorescence and phosphorescence spectra of *l*-CzTRZ in toluene, measured at 77 K.

6. Horizontal dipole orientation

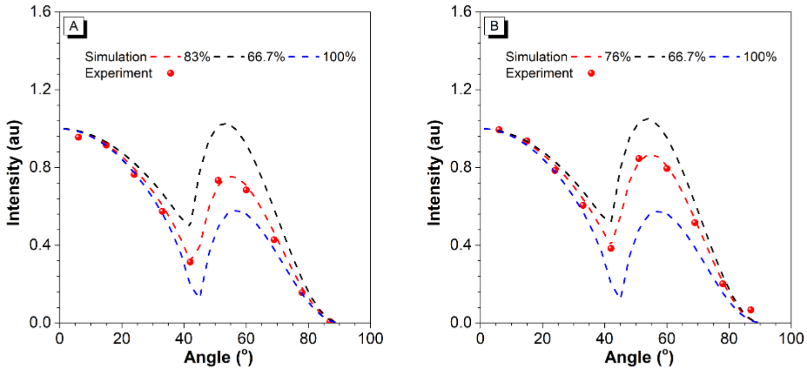

Figure S5. The *p*-polarized PL intensity of doped films of A) 3 wt% Ir(piq)$_2$acac: *l*-CzTRZ and B) 3 wt% PO-01-TB: CBP as a function of the emission angle.

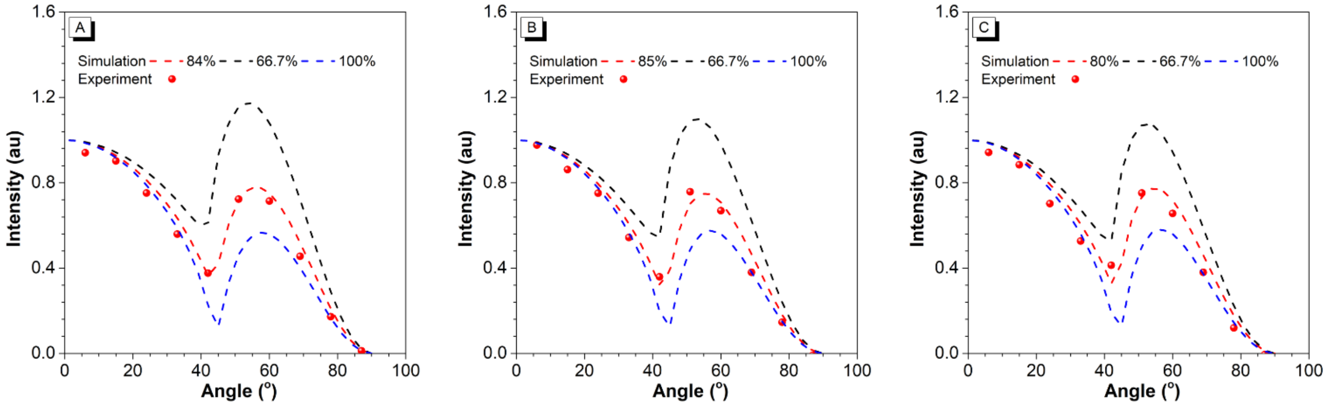

Figure S6. The *p*-polarized PL intensity of (A) the linear polar analogue in neat film, and the doped films of 3 wt% PO-01-TB: linear polar analogue and (C) 3 wt% $Ir(piq)_2acac$: linear polar analogue as a function of the emission angle.

7. OLED fabrication and measurement

Glass substrates precoated with a 90 nm layer of indium tin oxide (ITO) with a sheet resistance of 15~20 Ω per square were successively cleaned in ultrasonic bath of acetone, isopropanol, detergent, and deionized water, respectively, taking 10 minutes for each procedure. Then, the substrates were totally dried in a 70 ^{o}C oven. Before the fabrication processes, the substrates were treated by O_2 plasma for 10 minutes to improve the hole injection ability of ITO. The vacuum-deposited OLEDs were fabricated under a pressure of < 5 × 10^{-4} Pa in the Fangsheng OMV-FS450 vacuum deposition system. Deposition rate of organic materials, LiF and Al are 1~2 A s^{-1}, 0.1 A s^{-1} and 5 A s^{-1}, respectively. The luminance–voltage–current density characteristics and EL spectra were obtained via a PhotoResearch PR670 spectroradiometer, and an Ocean Optics USB 2000+ spectrometer, along with a Keithley 2400 Source Meter. The external quantum efficiencies were estimated utilizing the normalized EL spectra and the current efficiencies of the devices, assuming that the devices are Lambertian emitters. The effective emitting area of the device was 9 mm^2, determined by the overlap between anode and cathode. All the characterizations were conducted at room temperature in ambient conditions without any encapsulation, as soon as the devices were fabricated.

8. Reference

[1] Y.-J. Cho, A.-R. Lee, S.-Y. Kim, M. Cho, W.-S. Han, H.-J. Son, D. W. Cho, S. O. Kang, *Phys. Chem. Chem. Phys.* **2016**, *18*, 22921-22928.

Author Contributions

The manuscript was written through contributions of all authors. / All authors have given approval to the final version of the manuscript.
